# Comparison of the experience of caregiving at end of life or in hastened death: a narrative synthesis review

**DOI:** 10.1186/s12904-020-00660-8

**Published:** 2020-10-08

**Authors:** Jane Lowers, Melissa Scardaville, Sean Hughes, Nancy J. Preston

**Affiliations:** 1 Emory University, Palliative Care Center, 1821 Clifton Road, Suite 1016, Atlanta, GA 30329 USA; 2 American Institutes for Research, Atlanta, USA; 3 Lancaster University, Lancaster, UK

**Keywords:** Caregivers, Suicide, assisted, Grief, Motivation, Systematic review

## Abstract

**Background:**

End-of-life caregiving frequently is managed by friends and family. Studies on hastened death, including aid in dying or assisted suicide, indicate friends and family also play essential roles before, during, and after death. No studies have compared the experiences of caregivers in hastened and non-hastened death. The study aim is to compare end-of-life and hastened death caregiving experience using Hudson’s modified stress-coping model for palliative caregiving.

**Method:**

Narrative synthesis of qualitative studies for caregivers at end of life and in hastened death, with 9946 end-of life and 1414 hastened death qualitative, peer-reviewed research articles extracted from MEDLINE, CINAHL, Web of Science, and PsycINFO, published between January 1998 and April 2020.

**Results:**

Forty-two end-of-life caregiving and 12 hastened death caregiving articles met inclusion criteria. In both end-of-life and hastened death contexts, caregivers are motivated to ease patient suffering and may put their own needs or feelings aside to focus on that priority. Hastened death caregivers’ expectation of impending death and the short duration of caregiving may result in less caregiver burden. Acceptance of the patient’s condition, social support, and support from healthcare professionals all appear to improve caregiver experience. However, data on hastened death are limited.

**Conclusion:**

Caregivers in both groups sought closeness with the patient and reported satisfaction at having done their best to care for the patient in a critical time. Awareness of anticipated death and support from healthcare professionals appear to reduce caregiver stress. The modified stress-coping framework is an effective lens for interpreting caregivers’ experiences at end of life and in the context of hastened death.

## Background

End-of-life caregiving frequently is managed by friends and family, with an estimated 900,000 older adults receiving care from 2.3 million caregivers in the United States (U.S.) in 2011 [1], and rates of at-home death in Europe at 20–30% [2]. Caregivers face challenges in managing patients’ needs and household tasks, financial strain, and their own stress, anxiety, and exhaustion, among others, over the course of weeks or months [3]. Other studies have noted that end-of-life caregiving also carries potential for rewards in terms of meaningfulness and opportunities for closeness with the patient before death [4].

Multiple systematic reviews have examined the experiences and needs of end-of-life caregivers [3, 5–12]. Although many individual studies are rooted in conceptual or theoretical frameworks [13], few systematic reviews have them as an analytic structure: Broady [8] used personal construct psychology as the basis for framework analysis, and Morgan [10] conducted a feminist quality appraisal of gender in family caregiving. In general, systematic reviews identify common concepts of physical and emotional strain, stress, and feelings of helplessness; commitment, meaning making, and satisfaction; and the need for support and information [3, 5, 6, 8].

Hastened death through aid in dying, including assisted suicide and euthanasia, is available in parts of Europe, North America, and in Colombia. To date, one systematic review evaluated the experience of caregivers specifically in the context of aid in dying (including assisted suicide and euthanasia) [14]. Studies of caregiving during assisted dying describe caregiving roles such as helping the patient navigate the medical and legal hurdles to obtaining a lethal prescription, assisting with preparation of the medication, bearing witness to the death, and orchestrating the completion of patients’ wishes before, during, and after death [15–20]. Comparing the experiences of caregivers in aid in dying with those in other end of life trajectories can inform practice for clinicians supporting patients and caregivers before, during, and after hastened death. To date, no studies have directly compared hastened death with end of life caregiving.

This review uses Hudson’s [4] conceptual model of family caregiving for palliative care, which is based on Folkman’s [21] stress-coping model and seeks to draw a comparison of caregivers’ experiences during hastened and non-hastened death. In the stress-coping model, caregivers confronted with an event, such as a patient’s return home after a hospital stay, first appraise the event. Events seen as a threat, challenge, or harm are met with some coping strategy, either problem-focused or emotion-focused. The event outcome may be favourable or unfavourable, and the emotional outcome may be positive, distressing, or some form of meaning-based coping that informs future appraisals and coping approaches. Additionally, variables such as caregivers’ sense of preparedness or the patient’s disease status may mediate or moderate coping and emotional responses. (See Appendix 1 for definitions of model components.)

Applying the model in end-of-life and hastened death literatures separately facilitates development of a rich synthesis of caregiving within each context on its own and provides a rubric for comparing them. Further, themes identified inductively in either set of studies can provide insight into the strengths and limitations of the model itself.

## Methods

This narrative synthesis is rooted in constructionism and supposes that study participants, and researchers, build meaning and shape reality through their interactions with the world and with others. These created meanings are reflected in Hudson’s conceptual model, in which caregivers identify, appraise, and respond to events based on their own strengths or challenges.

Narrative synthesis can integrate diverse data against a framework or theory [22] and is useful for exploring heterogeneity across multiple studies [23]. This review follows Popay’s [22] recommended steps:

- developing a theory (in this review, Hudson’s model is the theory)
- developing a preliminary synthesis
- exploring relationships in the data
- assessing the robustness of the synthesis

### Review question and literature search

The review question, “What are the experiences of family and friends providing care at home for a person at the end of life or in the context of the patient’s hastened death?” can be broken into clearly defined population, exposure, context, outcome, and study design (PECOS) criteria [23, 24] listed in Table 1.

**Table 1:** PECOS Criteria

Population	Family members or caregivers of adult patients with life-limiting illness, through the point of death
**Exposure**	Caring for an adult patient who is dying (life expectancy < 3–6 months) or who chooses hastened death (medical aid in dying, voluntarily stopping eating and drinking, euthanasia)
**Context**	Caregiving in the home
**Outcome**	Caregivers’ emotional, practical, and philosophical experiences with caring for loved ones at end of life, either because of illness or related to deliberately hastened death
**Study Design**	Qualitative: interviews, focus groups, phenomenology, ethnography

The review question further may be broken down into a series of subquestions that align with Popay’s steps as follows:

- What are the experiences of caregivers for patients at end of life? (preliminary synthesis)
- What are the experiences of caregivers of patients electing hastened death? (preliminary synthesis)
- In what ways are caregivers’ experiences similar or different at end of life vs hastened death? (exploring relationships in the data)
- In what ways does the qualitative literature on end-of-life and hastened death caregiving support or refute Hudson’s model of caregiving experience? (assessing the robustness of the synthesis)

The review included two sets of searches of Medline, CINAHL, Web of Science, and PsycINFO — one for general end-of-life caregiving and one for hastened death. The Boolean search terms are described in Table 2.

**Table 2:** Key Search Terms

	End of Life	Hastened Death
**Population**	(Terminal* OR end-of-life* OR life-limiting OR cancer OR palliative OR hospice) AND (famil* OR caregiv*)	(Terminal* OR end-of-life* OR life-limiting OR cancer OR palliative OR hospice) AND (famil* OR caregiv*)
**Exposure (for hastened death searches only)**	N/A	[[(aid* OR assist*) AND (dying OR suicide)] OR [hasten* death] OR euthanasia OR [wish AND (hasten death OR die)]
**Context**	Home	Home
**Outcome**	Belief* OR experienc* OR emotion* OR support* OR need*	Belief* OR experienc* OR emotion* OR support* OR need*
**Study Design**	Qualitative	Qualitative

For parity between the two sets of data, the searches were limited to studies published between 1998, the year medical aid in dying was legalised in Oregon, the first U.S. jurisdiction to explicitly allow it, and April 2020. Searches were limited to peer-reviewed literature published in English involving human subjects. Additional studies were identified through citation tracking in relevant systematic reviews identified in the search process and in studies selected for inclusion.

### Selection criteria

Predefined inclusion and exclusion criteria (Table 3) guided title and abstract review of the initial results of each search and were the same for both searches. In addition, full-text searching omitted studies in which caregivers’ experiences could not be separated from those of patients or professionals, or studies in which current and former caregivers’ experiences were interwoven.

**Table 3:** Inclusion and Exclusion Criteria

Inclusion Criteria	Exclusion Criteria
Research published in peer-reviewed journals	Ethical or legal reviews
Published in English	Not about caregiver experience
Hospice or palliative care	Case reports, personal essays
Life expectancy < 6 months	
Patient has died, caregiver is bereaved	Animal studies
Qualitative, interview-based studies	Patients under age 18
Patient elected hastened death (hastened death review only)	Quantitative
	Patient elected hastened death (end of life review only)

All studies selected for full text review were reviewed using the Relevance, Appropriateness, Transparency, and Soundness (RATS) Quality/Appropriateness Appraisal Tool [25] to identify studies with limitations, such as unspecified recruitment or analysis methods that could warrant concern about the validity of the findings.

### Analytic approach

**Fig. 1:**
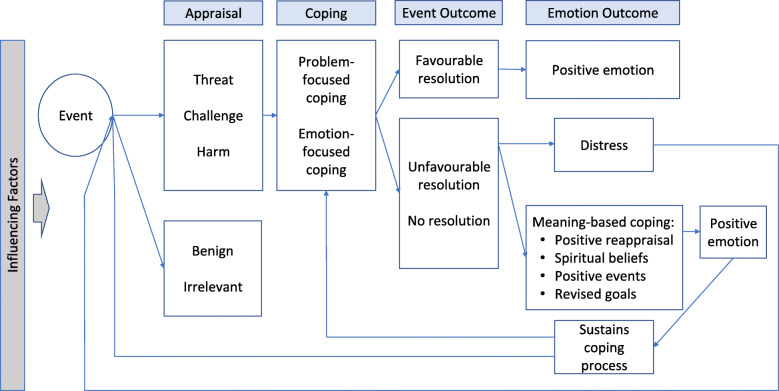
Modified Stress-coping Framework, modified from Hudson (2003)

Using Popay’s [22] narrative synthesis approach, participant narratives and author analysis in all studies in both searches were coded first in NVivo (QSR International) using thematic analysis to identify codes that fit within a priori themes aligned with elements of Hudson’s model (such as appraisal, coping, and event outcome, See Appendix 1: A Priori Codes), and subsequently using inductive codes representing concepts not found in the model (See Fig. 1).

Each code was analysed separately in each data set, employing subcodes where needed to clarify multiple concepts (for example, a favourable resolution could be getting needed services or the patient having a peaceful death). The two pools of studies were then synthesised individually within each theme of Hudson’s model as well as themes constructed outside the model. The two synthesised data sets then were analysed side by side to identify commonalities or differences. Where particularly illustrative, quotes are included.

## Results

**Fig. 2:**
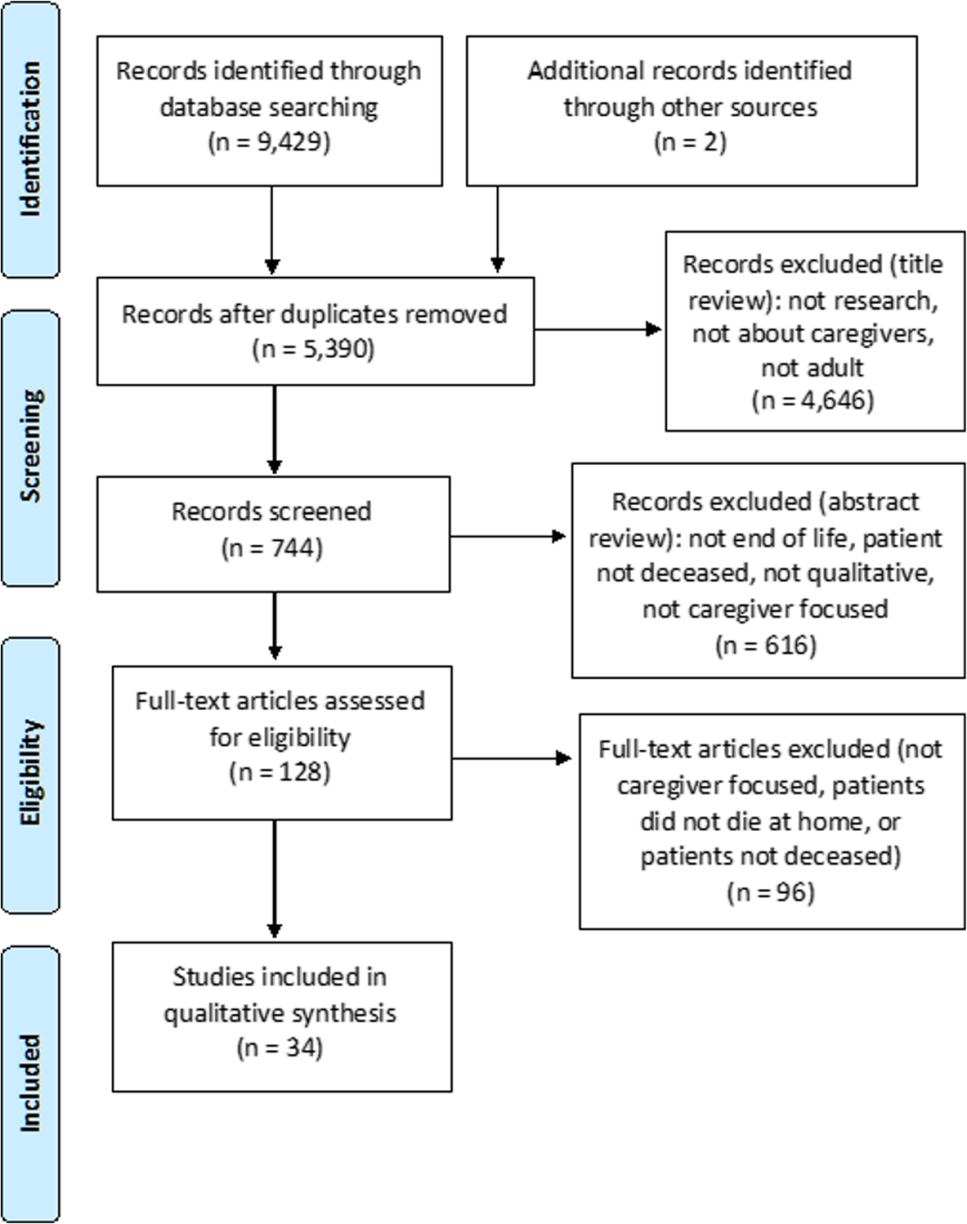
PRISMA (Preferred reporting items for systematic reviews and meta-analysis) flow chart describing the search process for end of life caregiving

A search using the end-of-life caregiving term set (See Table 2) yielded 9946 studies for review, with 5390 remaining after duplicates were removed (see Fig. 2). Two authors (JL, MS) scanned the first 10% of titles independently and conferred to refine the inclusion and exclusion criteria (see Table 3). After title review, 777 studies remained for abstract review. The two authors again assessed the first 10% of abstracts independently and conferred to further refine the inclusion/exclusion criteria. Studies were limited to those capturing experiences of bereaved caregivers who had cared for a patient through death at home. Following abstract review, 140 studies remained for full-text review; 40 studies met inclusion criteria. Finally, JL performed a manual review of studies included in relevant systematic reviews to search for possible overlooked studies, identifying two more and bringing the total to 42 (See Fig. 2).

**Fig. 3:**
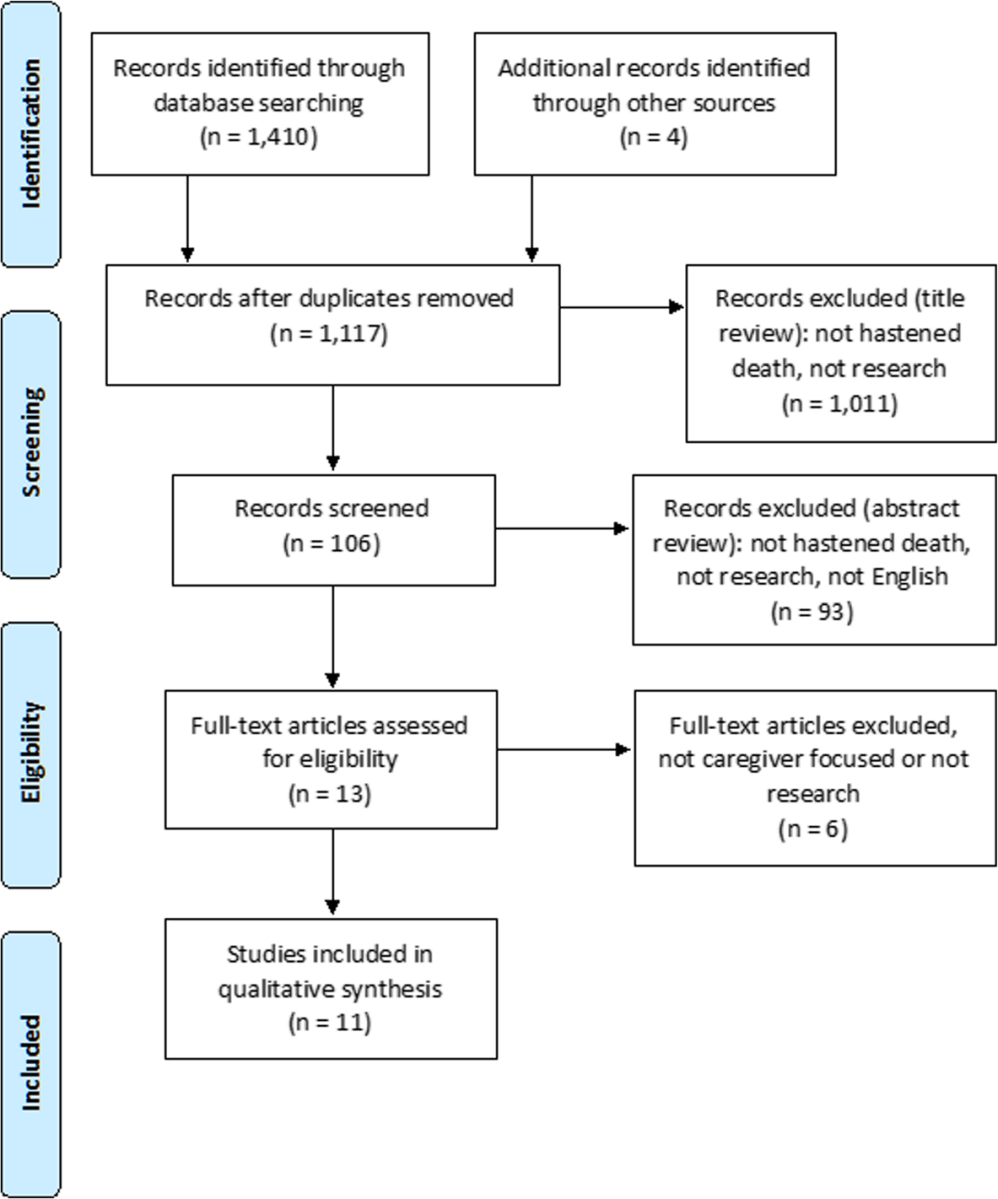
PRISMA (Preferred reporting items for systematic reviews and meta-analysis) flow chart describing the search process for hastened death caregiving

Searches using the hastened death term set (See Table 2) followed the same review process, yielding 1414 studies for review, with 1117 remaining after duplicates were removed. One hundred nine studies remained after title review, 13 after abstract review, and seven after full-text review. Hand-searching of references from those studies and relevant systematic reviews yielded five more, for a total of 12 (See Fig. 3).

### Overview of included studies

Of the 34 end-of-life caregiving studies, six were from Australia, seven from Canada, five from the U.S., four from the United Kingdom, 10 from elsewhere in Europe, one from Japan, and one from New Zealand (Table 4). Among the 12 hastened death studies, five were from the U.S., four from the Netherlands, two from Switzerland, and one from Canada (Table 5). Across both sets, cancer was a frequent cause of patient death, along with motor neurone diseases. Tables 4-5 list studies included in the syntheses; findings from studies are presented according to components of Hudson’s model in Tables 6-10.

**Table 4:** End of Life Caregiving Studies

Authors	Study design	Number of Caregivers	Patient Condition
Angelo, J 2014 [26] New Zealand	Phenomenology	6	Not specified
Aoun, SM 2012 [27] Australia	Thematic analysis	16	motor neurone disease
Armstrong MJ, 2019 [28] United States	Qualitative descriptive	30	Dementia with Lewy bodies
Bentley, B, 2016 [29] Australia	Thematic analysis	12	motor neurone disease
Carlander, I, 2011 [30] Sweden	Descriptive	10	Not specified
Cipolletta, S 2015 [31] Italy	Phenomenology	13	motor neurone disease
Clukey, L, 2007 [32] USA	Phenomenology	22	Cancer, heart disease, chronic obstructive pulmonary disease, hepatitis
Clukey, L, 2008 [33] USA	Thematic analysis	9	Not specified
Coristine, M, 2003 [34] Canada	Content analysis	18	Breast cancer
Dobrina, R, 2016 [35] Italy	Descriptive phenomenology	114	Cancer
Dumont, I, 2008 [36] Canada	Content analysis	18	Cancer
Fisker, T, 2007 [37] Denmark	Phenomenology	8	Not specified
Glass, AP, 2016 [38] USA	Case study	28	Alzheimer’s
Grbich, CF, 2001 [39] Australia	Thematic analysis	12	Cancer
Hasson, F, 2010 [40] Northern Ireland	Content analysis	15	Parkinson’s disease
Hasson, F, 2009 [41] Northern Ireland	Thematic analysis	9	Chronic obstructive pulmonary disease
Hisamatsu M, 2020 [42] Japan	Grounded theory	13	Cancer
Hovland CA, 2019 [43] USA	Content analysis	36	Dementia
Hughes, M, 2015 [44] Australia	Thematic analysis	28	Not specified
Johnson, A, 2003 [45] Australia	Narrative exemplars	1	Not specified
Kalnins, I, 2006 [46] Latvia	Phenomenology	18	cancer, stroke, heart disease
Linderholm, M, 2010 [47] Sweden	Hermeneutic analysis	14	Cancer
Lyckhage, ED, 2013 [48] Sweden	Phenomenological	6	Not specified
Mangan, PA, 2003 [49] USA	Constant comparison	15	Cancer
Mohammed, S, 2018 [50] Canada	Grounded theory	61	Cancer
Mori, H, 2012 [51] Japan	Framework analysis	34	Cancer
Ortega-Galán, 2019 [52] Spain	Phenomenology	81	Not specified
Payne, S, 2015 [53] England	Cross-sectional	59	Cancer, other
Robinson, C, 2017 [54] Canada	Constant comparison	29	Cancer
Sheehy-Skeffington, B, 2014 [55] Ireland	Thematic content analysis	16	Cancer, heart failure
Sinding, C, 2003 [56] Canada	Grounded theory	12	Breast cancer
Stajduhar, KI, 2013 [5] Canada	Secondary analysis of qualitative data	114	Not specified
Stone, AM, 2012 [57] USA	Constant comparison	35	Lung cancer
Strang, VR, 2003 [58] Canada	Not specified	15	Cancer
Strauss S, 2019 [59] USA	Discourse analysis	46	Not specified
Thomas, C, 2018 [60] England	Cross-sectional	30	Cancer, other
Totman, J, 2015 [61] England	Framework analysis	15	Cancer
Turner, M, England [62] England	Secondary analysis	17	Cancer, other
Vachon M, 2020 [63] Canada	Phenomenology	22	Not specified
Warrier MG, 2019 [64] India	Thematic analysis		Motor neuron disease
Wong, WK, 2009 [65] Australia	Thematic analysis	23	Cancer
Wu MP, 2020 [66] Taiwan	Grounded theory	22	Not specified

**Table 5:** Hastened Death Caregiving Studies

Author	Study Design	Type of Hastened Death	Number of Caregivers	Patient Condition
Albert, SM, 2005 [67] United States	Not specified	Patient wish for hastened death	80	Amyotrophic lateral sclerosis
Back, AL, 2002 [15] United States	Grounded theory	Physician-assisted suicide	35	Cancer, AIDS, neurologic, other
Buchbinder, M, 2018 [17] United States	Ethnography	Medical aid in dying	19	Not specified
Buchbinder, M, 2018 [18] United States	Grounded theory	Medical aid in dying	34	Cancer, amyotrophic lateral sclerosis
Dees, 2013 [68] Netherlands	Thematic analysis	Euthanasia	31	Cancer, neurologic, other
Gamondi, C, 2015 [19] Switzerland	Grounded theory	Assisted suicide	11	Not specified
Gamondi, C, 2018 [20] Switzerland	Grounded theory	Assisted suicide	11	Cancer, AIDS, neurologic, other
Georges, JJ, 2007 [69] Netherlands	Statistical analysis of interview data	Euthanasia or physician-assisted suicide	87	Cancer, amyotrophic lateral sclerosis
Holmes, S, 2018 [70] Canada	Content analysis	Medical assistance in dying	18	Cancer, organ failure, neurologic
Jansen-Van Der Weide, MC, 2009 [71] Netherlands	Secondary analysis of interview data	Euthanasia	86	Cancer, other
Snijdewind, MC, 2014 [72] Netherlands	Inductive analysis	Euthanasia or physician-assisted suicide	26	Cancer, old age, neurological
Starks, H, 2007 [16] United States	Inductive analysis	Hastened death	48	Not specified

**Table 6:** Appraisal

Appraisal	End of Life	Hastened Death
Benign	The patient is content and comfortable [26, 32, 36, 45, 53]	The patient receives services that facilitate their goal of hastened death. The death is peaceful [17, 18, 71].
Challenge	Coping with escalating number and intensity of caregiving tasks, patient’s decline, disruption in routine. Demands consistent with caregiver’s sense of duty and commitment, but achievable [26, 30, 37, 38, 45, 47, 48, 53–58, 60, 64].	Planning and preparation, reconciling one’s own beliefs to help the patient [15, 16, 68, 70]
Threat	Events that could affect the patient’s well-being, either internal (caregiver’s own preparedness and resources) or external (availability of services). Events that affect caregiver’s effectiveness, such as fatigue. Realisation of potential for death [27, 33, 36, 37, 42, 45–51, 57, 58, 60–64, 66].	Patient denied access to hastened death; risk of incomplete ingestion, difficult or prolonged death, legal repercussions after death, social stigma [15, 16, 18–20, 68–72]
		“*He started taking it and apparently it tastes awful, and so started gagging a little bit, and wanted to stop halfway. And we had discussed before, once you start it, you have to do the whole thing. So then we gave him alcohol. Ah, it was terrible...”* (Buchbinder et al., p. 5)
	*“And then you weren’t really sleeping because every few seconds you’re waking up and going ‘is she still breathing, is she still there?’”’ (Totman* et al*, p500)*	
Harm	Disease progression, insufficient professional help, potential to harm patient by being honest about prognosis [27, 29, 33, 36, 37, 41, 45–51, 56, 57, 61, 62]	Burden of secrecy about cause of death (Switzerland), inadequate support from providers resulting in more difficult death (U.S., Canada, Netherlands) [16, 19, 20, 68, 70]

**Table 7:** Coping

Coping	End of Life	Hastened Death
Problem focused	Solving logistical problems, learning new skills, keeping household running, arranging help, focusing on patient wishes, serving as gatekeeper [26, 30, 33–36, 38, 39, 45, 48, 46, 50, 51, 53–58, 65]	Planning and conducting logistics such as physician appointments or filling prescriptions, planning events before, during and after death, finding solutions for protracted or complicated dying [15, 16, 68, 70]
		*…caregivers offered practical support to assist patients with ingesting, such as getting juice or alcohol to chase the medication if the patient requested it, holding a cup, or keeping an eye on the time. Timekeeping was an important component of the process because patients were typically advised to ingest the medication quickly so as to avoid losing consciousness before finishing the lethal dose.* (Buchbinder et al. 2018, p4)
	*“So I remember us sitting down and then dividing the tasks, like, father doing the shopping, and my sister would do this, and I’d do that....” (Strang & Koop, p.110)*	
Emotion focused	Caregiving as an opportunity to show love, be rewarded with closeness; frustration, sadness, or anticipatory grieving [27, 32, 33, 36, 37, 42, 44, 45, 47, 51, 54–58, 60, 61, 63]	Overall focus on fulfilling patient’s desire to avoid prolonged suffering; where hastened death was illegal or quasi-legal, moral distress in trying to reconcile patients’ request for support with own ambivalence or discomfort. In Switzerland, carrying the burden of secrecy after death [16, 19, 20].
	“*So you know it was just a sadness that we couldn’t use the time to talk, to really, that I couldn’t help her prepare for her death.” (Sinding, p.158)*	
		*“My brother was used to say: “you do not have to be selfish, you do not have to think only for yourselves… if I want to do this thing is because I do not have solutions and I can’t bear it anymore.” Ehm…he was saying that we were selfish because we wanted to keep him alive… at all costs. Even in these conditions… so inhumane.” (Gamondi 2015, p149)*

**Table 8:** Event Outcome

	End of Life	Hastened Death
Favourable resolution	The caregiver has the skills and resources to solve a problem; death brings an end to suffering or is consistent with patient wishes; the caregiver has guidance or professional help in dealing with post-death tasks [28, 33, 36–38, 45, 46, 50, 54, 56, 57]	Healthcare providers help plan for or carry out the death; the caregiver finds the hastened death to be peaceful or joyful; loved ones have a chance for closure; the patient avoids unwanted suffering [15, 16, 66–70]
		“*We all toasted with the bourbon. Yep. And I mean, I haven’t been around many dying people so I don’t have experience with how that often goes, but this was joyful and peaceful, and it’s exactly what he wanted.” (Buchbinder* et al *p5)*
	‘*I feel maybe it’s hard to say but I knew the end would come and really it was a release not only for me but for X, I knew it was because it was very hard to watch him.’ (Hasson* et al *2010, p.733)*	
Unfavourable resolution	Professional help is unavailable or inadequate; the illness causes family tension; caregiving demands are unrelenting; the death is unexpected, and the caregiver feels unprepared [27, 34, 35, 42, 47–51, 53, 55, 56, 60]	Healthcare providers are unwilling to discuss hastened death; the patient cannot achieve hastened death and suffers; in Switzerland, the caregiver experiences ongoing distress about breaking social norms to assist in hastened death [15, 57, 70]
No resolution	Caregiver lives in state of constant vigilance; caregiver cannot process or mourn the patient’s death [32, 40, 51, 60, 61]

**Table 9:** Emotion Outcome

Emotion outcome	End of Life	Hastened Death
Positive emotion	Satisfaction with overall caregiving; patient’s serenity with own condition [45, 46, 49, 55–58]	Events that align with patient’s wishes [15, 17]
Distress	Patient decline, conflict between exhaustion and increasing patient needs, social isolation, breaking a promise to the patient, family conflict [27, 29, 30, 36, 37, 42, 45–51, 53, 56, 57, 60, 61, 63]	Complicated dying, moral distress about patient choice to die [15, 17–20, 67–71]
		*“The ‘I-killed-my-mom thing’ is big, still. Because it’s the truth—how do I come to some resolution around that?” (Starks* et al*, p117)*
	*“There’s a point where you’ve done, you’ve gone overboard. You hear the 110% effort stuff; well I think it’s probably 180% effort…. You just, you become a basket case.” (Sinding, p.157)*	
Positive reappraisal	Caring provides opportunity for growth, respect, closeness, or strengthening family ties. Death allows patient to escape suffering. Escalating need for care results in more clinical resources [26, 36–38, 44, 46, 48, 51, 53–57, 60–63, 65].	Clinicians who would not facilitate hastened death but were supportive in other ways; in retrospect, hastened death seen as right choice [15, 16, 18, 19, 70]
	*“I mean it’s so wonderful that you can give someone yourself. I mean that’s a real thing to do. And that they’ll let you.” (Sinding, p. 157)*	
Revised goals	Reducing hopes for patient’s future, deciding to encourage the patient to “let go” to avoid further suffering, admitting patient needs institution-based care [27, 28, 30, 32–34, 37, 38, 45, 46, 48, 53, 56, 59–63]	Putting own grief or ambivalence on hold to focus on patient’s wishes, reconciling to idea of hastened death as better option than disease trajectory or unassisted suicide [15, 18, 19]
	*‘I had to realize that this person was no [longer] capable mentally or physically, and I had to take over the role of [parent] just like you do, first it was like a 6 year old and then a 5 year old.’ (Clukey 2008, p312)*	
Spiritual beliefs	Taking comfort in a larger force to supply strength or determine patient’s fate, taking comfort in an afterlife [27, 32, 33, 36, 40, 56, 58, 59, 61]	Spiritual or ritual elements, during or after death, add to closure [16, 18, 19, 68]
Positive events	Events that eased suffering, allowed for closure, or provided humor [26, 32, 33, 36, 50, 55]	In U.S. and Canadian studies, deaths were described as joyful, sacred, or peaceful, with patients’ wishes achieved [16, 68, 70, 71].

**Table 10:** Influencing Factors

Influencing factors	End of Life	Hastened Death
Ability (preparedness, mastery, competence, self-efficacy)	Knowing what to expect, being prepared for patient’s death, feeling able to learn skills to meet new demands, taking pride in ability to care, having relevant previous experience [26, 27, 29, 30, 32–38, 40, 41, 43–59, 61, 62, 65, 66]	Because caregivers had not facilitated hastened deaths before, few reported ability-related factors. Not knowing how to manage a difficult hastened death was stressful [16, 17].
		“*I guess the only thing I wish is I think it would have been easier if we could have had more knowledge as far as how to do it; it would have been a whole ton smoother. And it ended up feeling fairly desperate. ...I don’t remember it as being anything negative, I just remember it as being exhausting.” (Starks* et al *p.117)*
	*“[Home palliative care physician] sat me down at one point, I think the last visit before she died…. He told me what I might expect and… That was invaluable.” (Mohammed* et al *p1232)*	
Structure (social support, information, respite)	Lack of support from friends and family, and lack of information about what to expect in caregiving were closely related to caregiver isolation and exhaustion. Caregivers acknowledged the importance of respite, but more often in retrospect after death [26, 28, 29, 32–42, 44, 46–51, 54–62, 64, 66]. *“In retrospect*. *.. my sister should have been trained, or somebody, to actually watch me for two weeks. .. you need to watch that caregiver and make sure she’s getting sleep and actually has her wits about her.” (Mangan* et al*, p252)*	Experience varied by jurisdiction: Swiss caregivers and U.S. caregivers where aid in dying was illegal reported feeling isolated by potential social stigma. Where hastened death was legal, some caregivers found support from family and friends. Swiss caregivers appeared to have adequate information about hastened death, but U.S. caregivers did not always have information on how to handle difficult deaths. Respite was not mentioned in hastened death studies [15, 19, 20, 69].
		*“The impossibility to tell “look, he has died of assisted suicide…” it was tremendous, it was sad.” (Gamondi* et al *2015, p. 150)*
Satisfaction (rewards, meaningfulness, mutuality, choice and commitment)	*Enhancing*: fulfilling sense of duty, showing love, meeting patient’s wishes, personal growth, being close with patient	*Enhancing*: being able to help patient enact wishes, being present for aided death, helping avoid suffering, taking place in sacred or celebratory event, engaging in communal act of planning and conducting death [16, 20, 70]
	*Challenging*: feeling inadequate when unable to meet all patient needs, needing to respect patient’s perspective [26, 27, 29, 32–41, 44–51, 53–66]	
		*“When I got down there that morning this whole circle of her closest people had done a ritual around this killing drug, this beautiful ritual around it.. .. They were all in a circle with a candle lit and they were emptying the capsules together and they were being playful and just the most beautiful energy, loving and making jokes and everything.. .. They prepared it in a very sacred and light way.” (Buchbinder 2018, p8)*
	*“I thought to myself, yeah, you’ve [wife] done things like that for me, it’s my turn to help you out and look after you and support you.” (Totman et al, p503)*	
Outlook (anxiety, depression, and psychological distress; positive emotion; optimism)	*Enhancing*: satisfaction with performing well, feeling appreciated, closure	Setting aside anticipatory grief to focus on patient, seeing patients achieve wish of peaceful death and release from suffering [17, 18, 70]
	*Challenging*: Impending loss of patient, relentless burden of caregiving, gradual loss of closeness with patient, not wanting to harm patient’s optimism [26, 30, 31, 35–37, 41, 44, 46, 48, 49, 53, 54, 56, 58, 60–65]	
Personal (cultural factors; caregiver burden and health; patient’s disease status, level of dependency, and duration of illness; caregiver age, gender, socioeconomic status)	Exhaustion from caregiving, balancing caregiving and other life responsibilities, sense of duty to patient, patient’s acceptance or denial of condition [26, 27, 29–40, 46–62, 64, 66]	Understanding patient’s current suffering, likely trajectory and the inevitability of death, shared expectation that hastened death would be more comfortable, lack of clarity about when hastened death would be appropriate [15, 70, 72]

In Hudson’s model, the process of appraisal, coping, and resolution begins with identification of an event. Caregiving at end of life was both a single overarching event and the sum of many smaller events. However, for end-of-life caregivers, events focused on changes in patients’ needs, whereas in hastened death, events primarily followed a predictable pattern of planning, preparation, orchestrating the death, and tying up loose ends. The results of the synthesis are presented in the context of Hudson’s model from appraisal through outcome, followed by influencing factors, and lastly by inductive themes not represented within the model.

### Themes from the literature review using a priori themes from Hudson

#### Appraisal (Hudson)

Appraisal is the caregiver’s initial assessment of the environment (or an event) and whether it falls within or beyond the caregiver’s resources. In studies on end-of-life caregiving, events appraised as irrelevant rarely merit mention in final study analysis (Table 6). End-of-life caregiving events appraised as benign include those in which the patient appeared content and comfortable, such as having guests or being bathed. For both sets of caregivers, challenging events were those that tested caregivers’ capacity but were important to carrying out their commitment to caring for the patient. For end-of-life caregivers, coping with escalating care needs despite fatigue was challenging; reconciling their own ambivalence to aid in dying challenged hastened death caregivers.

Among end-of-life caregivers, events perceived as threats primarily concerned the patient’s well-being and could be internal (the caregiver’s own preparedness and resources) or external (unavailability of hospice or other support). However, caregivers also perceived threats to themselves, such as the toll of fatigue or conflicts from other family members with differing views of care goals; or threats to the family, such as exposure to the patient’s deterioration. Hastened death caregivers primarily identified threats as things that jeopardised patient’s ability to achieve his/her desired death: uncooperative physicians, incomplete ingestion of lethal medication, or a difficult or prolonged dying process. For hastened death caregivers, the possibility of legal consequences following the death and the potential for social stigma, particularly in Switzerland, were threats to their own well-being before, during, and after the death.

End-of-life caregivers identified multiple sources of harm, including disease progression, insufficient professional care, and the potential that being honest about prognosis would be detrimental for the patient. In hastened death studies in Canada and the U.S., events appraised as harms were those in which health professionals caused the patient to suffer more than necessary by making hastened death more difficult.

#### Coping (Hudson)

Coping includes the caregiver’s thoughts, feelings, and actions in response to appraisal. In both sets of studies, the logistical demands of caregiving require frequent problem-focused coping, but the overarching activity of caregiving appears motivated by emotion and concern for the patient (Table 7). Anticipatory grief is common among end-of-life caregiving studies but rarely discussed in hastened death studies. Rather, hastened death caregivers described setting their own feelings aside for the finite time left to focus on patient needs.

#### Event outcome (Hudson)

Event outcomes are the caregiver’s appraisal of whether the event’s results are consistent with his/her goals. Caregivers in both groups frame their views on death in terms of the patient’s wishes – such as avoiding suffering – regardless of their own feelings (Table 8). In end-of-life studies, positive events are those that involve the patient’s status, whereas events can be viewed as unfavourable if they have negative consequences for either the patient or caregiver. In most studies, hastened death caregivers tend to view events in terms of the patient’s goals rather than their own needs.

#### Emotion outcome (Hudson)

Emotional outcome is the caregiver’s reaction to the event outcome. In Hudson’s model, it can include positive emotion or distress, but also different types of meaning-based reframing, such as setting revised goals, that can inform future appraisal and coping. Being reconciled to the patient’s death and helping the patient avoid unnecessary suffering were tied to positive emotional outcomes or the ability to reframe events positively for both sets of caregivers (Table 9). End-of-life caregivers who were unprepared for the death found caregiving more distressing, and the patient’s suffering also caused distress for both groups. The grueling nature of long-term caregiving also was distressing for end-of-life caregivers, particularly when circumstances led to a feeling of letting the patient or family down. For some hastened death caregivers, the intentionality of the death led to distress. Thus, for both sets of caregivers, a feeling of violating family or cultural expectations about dying and caregiving led to distress.

### Influencing factors

Hudson lists 18 variables that can influence caregivers’ experience (see Appendix 1 for definitions). Although each is distinct and based on other research or conceptual models, they can be broadly clustered as:

- Ability (preparedness, mastery, competence, self-efficacy)
- Structure (social support, information, respite)
- Satisfaction (rewards, meaningfulness, mutuality, choice and commitment)
- Outlook (anxiety, depression, and psychological distress; positive emotion; optimism)
- Personal (cultural factors; caregiver burden and health; patient’s disease status, level of dependency, and duration of illness; caregiver age, gender, socioeconomic status)

Caregiver age, gender, and socioeconomic status were excluded from this analysis because they were not possible to tease apart in a synthesis of multiple published works.

End-of-life caregiving studies had ability-related codes more often than hastened death studies, possibly because the duration of end-of-life caregiving facilitated learning new skills or gaining confidence in abilities (Table 10). Hastened death caregiving, by contrast, was a one-time process with few steps repeated and little precedent. End-of-life caregivers frequently described exhaustion and mentioned the value of respite, but hastened death caregivers did not, perhaps because of the shorter timeframe or a choice to defer their own needs until after the death.

Many influencing factors could be positive or negative. Social isolation and lack of information were stressful for both end-of-life and hastened death caregivers. Meeting the patient’s wishes was related to satisfaction in both groups, while being unable to meet expectations for care was stressful. Hastened death caregivers, particularly in the U.S. and Canada, often described preparing for the death as communal, and the death itself as sacred or beautiful, whilst those in Switzerland were more likely to describe fear of stigma if the cause of death were widely known.

Most factors identified in Hudson’s model could either enhance caregivers’ experience or create additional challenge. For example, patients who spoke openly about their own decline and impending death relieved caregivers of feeling a need to protect the patient from the truth or hide their own acceptance of the coming death; end-of-life caregivers for patients in denial expressed distress about not wanting to dash the patient’s hopes.

Healthcare professionals are not listed as an influencing factor in Hudson’s model, but their role is a frequent theme in caregiving studies, either as sources of support and information or representing failures of the health system to adequately respond to patient and caregiver needs (Table 11).

**Table 11:** Healthcare Professionals

	End of Life	Hastened Death
Healthcare professionals	*Enhancing*: providing instruction and information, handling tasks beyond caregiver’s skill, acknowledging caregiver effort, providing regular social interaction or respite	*Enhancing*: providing information about what to expect in death
		*Challenging*: lack of comfort in discussing or supporting patient’s desire for hastened death [15, 16, 18–20, 69, 71]
	*Challenging*: lack of care coordination or continuity, lack of empathy, lack of specialised knowledge or services, lack of clarity about available services, focus only on patient, disappearance of services after death [27–37, 43, 46–54, 57, 58, 61, 62, 64]	

### Inductive themes: other factors

Beyond the themes outlined in Hudson’s model, other internal and external factors appear to affect caregivers’ experiences (Table 12). The structure of healthcare, nationally or locally, affects whether homecare services or hospice is available, whether specialised care for conditions such as motor neurone disease is available, and whether patients and caregivers can readily find out about services for which they are eligible. Costs of medication and equipment also can add to caregivers’ burden. For hastened death caregivers, whether hastened death was legal and whether information and support were available affected moral distress and preparedness to facilitate a comfortable death.

**Table 12:** Other Factors

Other factors	End of Life	Hastened Death
Structure of health care delivery	Availability, or not, of specialised services or at-home care support, cost of care, social policies supporting family caregiving [34, 41, 46, 49, 55, 61]	Legality, or not, of hastened death [15, 19, 20]
Grief	Variable acceptance of impending death, anticipatory grief [27, 31, 32, 36, 37, 41, 46–48, 51, 60]	Acceptance of hastened death as better than suffering or prolonged dying [19]

Caregivers reported different fundamental motivations for providing care. In addition to cultural norms and a desire for closeness at the end of the patient’s life, some end-of-life caregivers also expressed distrust of the healthcare system, particularly hospitals, as motivation to care for the patient at home. Whilst some caregivers saw the hospital as a fallback solution if the patient’s needs became too great, others saw the potential of sending the patient to the hospital as a sign that they had failed at caregiving.

Finally, grief affects caregiving at the end of life. Anticipatory grief was common among end-of-life caregivers. On the one hand, coming to terms with the patient’s impending death was associated with easier resolution of grief after death. On the other hand, the weight of anticipatory grief could lead caregivers to shut down their emotions or to seek distraction in the form of tasks. In this respect, grief might affect whether caregivers take a problem- or emotion-focused approach to events in either group.

## Discussion

This theory-centered review uses Hudson’s caregiving model [4] as a structure for synthesising results of studies that evaluated caregivers’ experiences in caring for patients at home at end of life and in the context of hastened death. Whilst many of the themes identified in analysis fit consistently with the model, themes constructed inductively and relationships across concepts suggest opportunities to refine the model:

### The role of healthcare professionals

Healthcare professionals play a major role in caregivers’ experience at end of life. Professionals provide knowledge, teach skills, take decision making pressure off the shoulders of caregivers, offer support and validation, and can be a gateway to resources [3, 6, 7]. When healthcare professionals are unavailable, do not fulfill promises to take measures to relieve patient suffering, or do not support caregivers’ assessment that the care is too much to handle, caregivers often report feeling isolated. Meta-analyses of caregiver studies noted that across many studies, caregivers expected health professionals to take responsibility for developing a trusting, supportive relationship with families [3, 6]. For some end-of-life caregivers, the regular presence of hospice staff is a welcome, regular break in caregiving, and its loss is felt after the patient’s death. In hastened death contexts, professionals’ legal ability or personal willingness to discuss the patient’s wishes and options, and provide practical support, contributed to caregivers’ reduced moral distress and increased satisfaction that the patient’s wishes could be achieved. The role of professionals is not highlighted in Hudson’s model but might fall into either social support or information.

### Healthcare policy

Whilst some caregivers reported having their needs anticipated well and addressed, others reported isolation, stress, and in some cases financial strain as the patients’ needs outstripped the support structures available [3]. For example, family caregivers for patients with motor neurone disease in Australia reported difficulty accessing community palliative care services or support adequate to the increasing demands of the disease [29]. These structural issues are distinct from the availability or attitude of individual health professionals and may be more relevant in countries with limited or inequitable healthcare infrastructure as opposed to national health coverage [11]. Categorised unmet needs in studies of palliative care patients receiving services at home included transportation, equipment, caregiving support, and respite, in addition to adequate communication and information from professionals. However, a systematic review of quantative studies of caregiver experience found a lack of consistent, high-quality evidence that specific services and programmes improve caregiver outcomes [13].

### Certainty of death

Acceptance and anticipation of patient’s death appears related to having less grief before and after death. In hastened death studies, caregivers are actively working toward the patient’s goal of a peaceful death, whereas some end-of-life caregivers are unprepared or surprised by the death. Hudson’s model is not end-of-life-specific, but grief may be a relevant factor for caregivers when death is likely. Broady’s [8] scoping review of caregiver literature notes that anticipatory grief may encompass awareness of both the patient’s impending death and the change in identity, away from caregiver, that will follow.

The analyses reveal similarities and differences between caregivers’ experiences in end of life and hastened death settings. Across studies, caregivers commonly sought closeness with the patient and reported satisfaction at having done their best to care for the patient in a critical time. Hastened death caregivers were more consistently reconciled to the patient’s death and the belief that death was preferable to anticipated suffering. Some but not all end-of-life caregivers reached this conclusion. However, the deliberate nature of hastened death may mean that patients choose likeminded caregivers more deliberately than in situations where caregiving may not be expected to lead to death [14]. Unlike in studies of end-of-life caregivers, physical exhaustion and burden were not commonly reported among hastened death caregivers [14], possibly because their scope of preparing for hastened death is finite.

### Strengths and limitations

This review is the first to apply Hudson’s model as a lens for synthesising literature on the experience of caregivers at end of life. As such, the review also evaluates the limits of Hudson’s model and identifies potential refinements, such as the role of healthcare professionals as an influencing factor, that could strengthen it.

A major limitation of synthesising qualitative studies against such a model is that they may use other analytic models that may emphasise different aspects of caregiving. Further, because each study represents data synthesised from participants by the authors, salient aspects of Hudson’s model, such as the appraisal of benign caregiving events, may have fallen out of the earlier published work in favour of events that better aligned with the authors’ own theoretical underpinnings. Applying Hudson’s model against a full set of original data may better illuminate its strengths and weaknesses.

Analysis is further limited by the unknown degree to which studies have accurately represented the experiences of participants; for example, whether caregivers in hastened death shared completely with interviewers their emotional response to the death. Limiting inclusion to English-language publications reduces the comprehensiveness of the hastened death analysis. Research on hastened death in the Netherlands, Belgium, Switzerland and other countries may be published in local languages. Future analyses could take additional steps to secure multilingual sources, including soliciting articles from other scholars in the field.

## Conclusion

In both end-of-life and hastened death contexts, caregivers are motivated by the desire to ease patient suffering and may put their own needs or feelings aside to focus on that priority. Hastened death caregivers’ expectation of impending death and the relatively short duration of caregiving may result in less caregiver burden relative to end-of-life caregivers. Acceptance of the patient’s condition, social support, and support from healthcare professionals all appear to improve caregiver experience. Hudson’s model is an effective lens for interpreting caregivers’ experiences at end of life and in the context of hastened death, although modifications such as the inclusion of professional caregivers could strengthen it.

## Appendix

**Table 13:** A Priori Codes from Hudson Conceptual Model of Family Caregivers for Palliative Care

Event	Change in environment or patient status, e.g., new information, worsening of symptoms, return home from hospital
Appraisal	Determining whether event is relevant to caregiver or patient’s well-being
Threat	Event poses a threat to patient or caregiver well-being that may be outside of caregiver’s capacity to address
Challenge	Event poses a potentially surmountable obstacle within caregiver’s capacity
Harm	Event leads to direct harm to patient or caregiver
Benign	Event is unlikely to change patient or caregiver status or may improve it
Irrelevant	Event has no bearing on patient or caregiver status
Coping	
Problem-focused coping	Acting on oneself or the environment, such as seeking information
Emotion-focused coping	Changing the relationship to the environment, or changing the relational meaning of the experience to avoid stress
Event Outcome	
Favourable resolution	Outcome is consistent with goals and values
Unfavourable resolution	Outcome is contrary to goals and values, such as harm
No resolution	Situation persists without opportunity for change
Emotion Outcome	
Positive emotion	Favourable resolution leads to satisfaction, end of coping
Distress	Unfavourable resolution of event leads to distress
Meaning-based coping	Unfavourable or no resolution leads to adapting one’s mental state to be able to respond to an event
Positive reappraisal	Finding meaning in the event based on beliefs and values
Revised goals	Adjusting goals for situation to obtain control
Spiritual beliefs	Activating spiritual beliefs to fuel emotion- or problem-based functions
Positive events	A satisfactory outcome to the event leads to positive appraisal
Variables	
Preparedness	How ready the caregiver perceives being, regardless of actual skill or knowledge
Mastery	Sense of control and enhanced self-esteem through overcoming a stressor, development of new abilities, very broadly (not task-specific)
Competence	Perception of self as adequate at caregiving specifically
Self-efficacy	Belief in one’s own ability to manage a situation. Not an inherent trait but event- and task-specific
Anxiety, depression and distress	Negative psychological effects of ongoing caregiving demands
Social support	Interactions with friends, family, coworkers. Can be positive or negative, or absent.
Information	Seeking information to assess problems and solutions. Successful information seeking facilitates more effective coping.
Rewards	Satisfaction, positive emotional gains from caregiving, such as receiving love from patient, seeing patient content, feeling accomplished
Meaningfulness	Caregiver sees role as worthwhile investment or challenge
Positive emotions	Feelings of happiness, satisfaction, recognition as opposed to stress
Optimism	Inherent trait that buffers caregiver against strains of caregiving
Mutuality	Gratitude and meaning and idea of reciprocity in relationship with patient, closeness
Respite	Activities or interactions outside of caregiving that reduce stress and allow caregiver to recognise his/her own needs and interests
Cultural factors	Expectations about familial roles that shape expectations of caregiving and influence stress and coping (e.g., duty or honour to care for spouse or parent)
Caregiver burden and health	Physical, emotional, psychological, financial, or social problems related to caregiving (e.g., lack of sleep, numbed emotions, isolation)
Choice and commitment	Making a conscious choice to take on caregiving role
Patient’s disease, dependency, and illness duration	Patient’s physical needs, psychological aspects of illness, and own recognition and outlook on illness
Caregiver age, gender, socioeconomic status	Unclear but possible relationships in response to caregiving based on relationship status, age (physical ability), economics
Additional codes	
External influences	Legal, economic, or other structural factors that shape the environment in which care is provided overall and the caregiver’s options for providing care (e.g., insurance, sick leave)
Grief	Anticipatory or posthumous grieving

## Abbreviations

AIDS: Acquired Immune Deficiency Syndrome
CINAHL: Cumulative Index to Nursing and Allied Health Literature
PECOS: Population, Exposure, Context, Outcome, and Study design
PRISMA: Preferred Reporting Items for Systematic Reviews and Meta-analysis
RATS: Relevance, Appropriateness, Transparency, and Soundness Quality/Appropriateness Appraisal Tool
